# Endangered shark species traded as “cação” in São Paulo during the COVID-19 lockdown: DNA-barcoding a snapshot of products

**DOI:** 10.1007/s11033-023-08876-6

**Published:** 2023-10-29

**Authors:** Veronica Zuccolo, Fernanda Moreira Rego, Emily Hughes, Andrew M. Griffiths

**Affiliations:** 1 Hatherly Laboratories, Department of Biosciences, University of Exeter, Prince of Wales Road, Exeter, Devon UK; 2 Independent Researcher, Santos, São Paulo Brazil

**Keywords:** DNA barcoding, Mislabelling, Shark, Endangered species, Fishing monitoring

## Abstract

**Background:**

Elasmobranch populations are declining, predominantly driven by overfishing, and over a third of global sharks, rays, and chimeras are estimated to be threatened with extinction. In terms of trade, Brazil is ranked the eleventh-largest shark producer and the top importer of shark meat in the world. Research has shown that elasmobranchs are sold in Brazil under the name “cação” (a generic designation for cartilaginous fish) to overcome consumer resistance.

**Methodology and results:**

This study used DNA barcoding to investigate the sale of sharks in the State of São Paulo during the COVID-19 lockdown. A total of 35 samples of “cação” were analysed, revealing six different shark species on sale, including *Carcharhinus falciformis*, *Carcharhinus signatus*, *Carcharias taurus*, *Isurus oxyrinchus*, and *Isurus paucus,* that are threatened with extinction according to the IUCN red list. This study demonstrates that vulnerable elasmobranchs are being commercialised under the label “cação” in the São Paulo State and Brazil.

**Conclusions:**

Comparison of shark products traded before and during the COVID-19 pandemic showed no significant difference, suggesting lockdown did not affect patterns of species commercialisation. Effective fisheries and sale monitoring, correct product labelling legislation and increased consumer awareness that “cação” is shark are needed for appropriate conservation and management of shark populations in Brazil.

**Supplementary Information:**

The online version contains supplementary material available at 10.1007/s11033-023-08876-6.

## Introduction

Since 1970, the abundance of oceanic sharks and rays has experienced a decline of 71%, which has been linked to an 18-fold increase in fishing pressures [1]. The result is that over a third of global Chondrichthyes are now threatened with extinction on the IUCN Red List [2]. Overexploitation, often driven by a demand for shark fins and meat, is considered the main cause of elasmobranch decline [2].

Brazil has the eleventh-largest capture rate for sharks globally and has a vigorous artisanal and commercial fishery with high levels of elasmobranch bycatch [3]. The country is also ranked as the largest importer of shark meat in the world [4]. Brazil has been recognised as one of the global hotspots for shark conservation [5], however, over 30% of all elasmobranch species in Brazil are at risk of extinction [6], with excessive fishing pressure named as the main contributor to these declines [2, 7]. More than a dozen shark species are caught as bycatch or targeted by the tuna longline fleet, with *Prionace glauca* (blue shark), *Isurus oxyrinchus* (shortfin mako) and *Carcharhinus falciformis* (silky shark) among the most commonly captured [8]. The elasmobranch capture rate in Brazil is also likely to be much higher than the official figures, due to issues with the accuracy of fisheries data including: the grouping of multiple shark species under a single designation, inconsistent monitoring of fishing vessels and the absence of a countrywide fisheries records for ten years [9–11].

In Brazil, shark meat is sold under the umbrella term “cação”, a generic designation for cartilaginous fish [12], designed to boost consumer acceptance [13]. The use of such a non-specific label helps to obscure the trade in elasmobranchs and hinders their conservation [14]. Surveys found over 70% of Brazilians were unaware that the term “cação” refers to sharks [15], showing low levels of consumer awareness [13]. Furthermore, 62% of people interviewed in Brazil considered shark meat to be of high quality, due to its pleasant taste, lack of bones and smoothness [16]. Elasmobranch meat is also regarded as relatively cheap [10]. Researchers and organisations in Brazil have made recent efforts to raise public awareness that “cação” is shark meat and to educate the population regarding mislabelling and its negative impact on shark populations [17].

DNA barcoding has become a widespread technique used for species identification, with the cytochrome oxidase I (COI) gene extensively used by researchers to identify processed shark products [18, 19]. Utilisation of DNA barcoding has uncovered mislabelling and trade of endangered elasmobranch products in many locations across the globe [20, 21], with an increasing focus on Brazil [12, 22, 23]. Two recent Brazilian studies using DNA barcoding found that 43% and 55% of their “cação” samples comprised of threatened species according to the IUCN red list, with the blue shark (*Prionace glauca*) identified as the most commonly traded species [24, 25].

The State of São Paulo, located in Southeast Brazil, is likely to be the largest importer and consumer of shark meat in Brazil [10] and previous investigations have highlighted the utilisation of endangered and/or prohibited species as part of the trade in “cação” here, typically by sampling carcasses and focusing on sharks of high conservation concern. One study included the application of multiplex PCR to investigate sharks of the genus *Carcharhinus*, where 48% of carcasses sampled at landing were identified as night shark (*Carcharhinus signatus*), protected in Brazil since 2004 [26]. Another focused on angelsharks and employed DNA barcoding, which identified *Squatina Guggenheim*, *Squatina occulta* and even the Brazilian guitarfish (*Pseudobatos horkelii*), all endangered species in Brazil [27]. Both studies highlighted the need for further investigation of the sale of prohibited and endangered species in São Paulo.

This study utilised the COI DNA barcoding to investigate the sale of shark meat products as “cação” in São Paulo State during the Coronavirus (COVID-19) pandemic lockdown to determine if endangered and prohibited species were being sold. To explore whether any changes in practice or reduced fisheries monitoring during the COVID-19 lockdown impacted patterns of species commercialisation, a comparison was also made to recent DNA barcoding investigations in Brazil conducted prior to the COVID-19 pandemic.

## Methods

### Sample collection

**Fig. 1:**
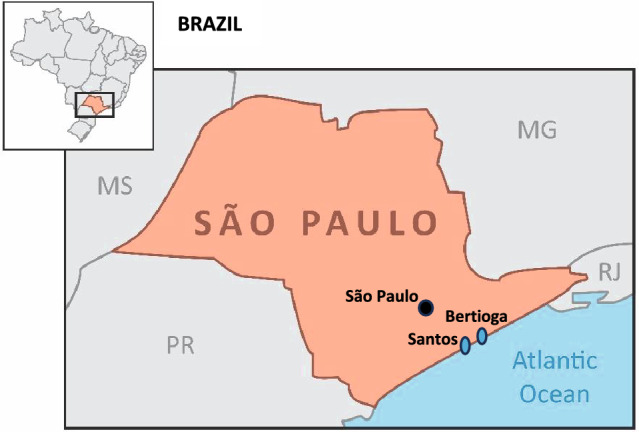
Map illustrating sample locations. Brazil shown in grey, and the State of São Paulo in orange. The black circle corresponds to the city of São Paulo, whilst the blue ovals indicates the coastal areas of Santos and Bertioga

A total of 35 samples of “cação” were obtained from 34 retailers (seafood wholesaler, fishmongers, and food markets) in São Paulo State, Brazil (cities of São Paulo, Bertioga, and Santos) (Fig. 1) between May–June 2020. Sample collection was conducted during the COVID-19 lockdown, uniquely permitting consideration of its effect on the commercialisation of threatened species. Local COVID-19 guidelines, including social distancing and lockdown measures, made the sampling significantly more challenging, consequently impacting the number of shark meat products obtained. Most of the samples (60%) were sold as fresh/unfrozen (Supplementary Material), however, discussion with sellers suggests freezing and thawing of products may occur in the wholesale chain, even in products marketed as fresh.

Small muscle tissue samples of < 25 mg (~ 1.0 cm^3^) were extracted from each sample and preserved in 1ml of RNAProtect (QIAGEN; Venlo, Netherlands) and stored at − 20 °C. The samples were shipped to the UK for molecular analysis at the University of Exeter (Exeter, UK).

### DNA extraction and sequencing

DNA was extracted from the tissue samples, following a HOTSHOT protocol [28]. The PCR amplification of ~ 650 bp of the COI region followed Serra-Pereira et al*.* [29] and Ivanova et al. [30]. Subsequently, on samples that failed to amplify, a shark specific multiplex of primers was attempted [18]. This combines primers for a full COI DNA barcode, alongside a mini-barcoding approach using forward primer that has been shown to be effective on degraded samples [31]. After successful PCR amplification, the products were sent to GENEWIZ (Takeley, UK) for purification and Sanger sequencing with the forward primer. Two samples were also sequenced using the reverse mini-barcode (M13R) to help distinguish between *Carcharhinus* species.

### Sequence and statistical analysis

The DNA barcodes generated were manually checked using BioEdit v7.2.5 [32] to remove the primer sequences and inspect the read quality. The sequences were referenced against GenBank [33] and BOLD [34]. This identified the top-match species corresponding to the sequences with > 98% homology. The conservation status of each species identified was referenced by consulting global [35] and national [7] extinction risk categories that were current at the time of data collection (i.e. ~ June 2020).

To explore the impact of COVID-19 lockdown on patterns of shark product commercialisation, a comparison was made between the data gathered here and two recent pre-pandemic DNA barcoding studies investigating the trade of shark species in Brazil. This includes the work of Merten Cruz et al. [23] who collected 55 shark samples along the Brazilian coastline in 2017, including 17 sourced in São Paulo State and Queiroz et al. [36] who collected 15 products from within São Paulo State in 2019. These represent the closest investigations in terms of geographic and temporal scope (that were conducted before lockdown), for comparison to this study. A one-way non-parametric similarity analysis (ANOSIM) using Bray–Curtis distance measure for each pre-pandemic study was conducted in Past v4 [37].

## Results

DNA barcodes were obtained from all 35 products (average length 238 base pairs, bp), with nine samples (25%) successfully sequenced with the long COI barcode (average length 584 bp). The remaining 26 products yielded mini-barcodes (average length 127 bp), with two samples also sequenced using the additional reverse mini-barcode. All barcodes provided top species matches above 98% identity on GenBank and BOLD, with an average identity of 99.9% to their top matching species (Supplementary Material).

These sequences enabled the successful identification of 35 “cação” samples to species level, including six species of sharks: *I. oxyrinchus, Isurus paucus* (longfin mako)*, P. glauca, C. falciformis, Carcharias taurus* (sand tiger shark) and *C. signatus*. For five samples, the barcodes generated had equal top matches to multiple records of *P. glauca* and a single *Carharodon carcharhias* record on Genbank. However, after reviewing the *C. carcharhias* sequence and conducting phylogenetic analysis (accession number JQ 654702.1, Supplementary Material), it was concluded that it was incorrectly identified on Genbank and discounted. It is important to note that the species determination of two samples (including *C. falciformis and C. signatus*) was only possible with the additional use of reverse mini-barcodes, helping to provide a top-match to a single species.

**Fig. 2:**
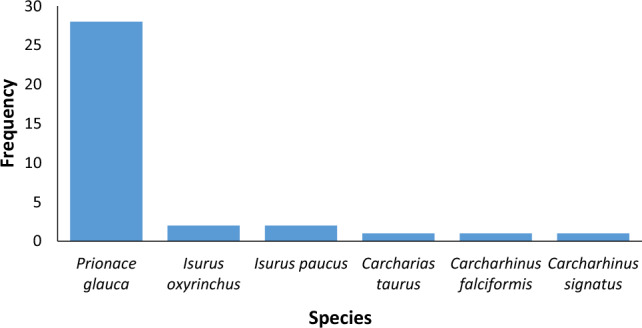
Bar chart of species identified (n = 35)

The most common species identified was blue shark (*P. glauca*, 28 products). The other shark species were less frequent with *I. oxyrinchus* and *I. paucus* identified in two products each, the remaining products were all identified to unique species (Fig. 2).

**Fig. 3:**
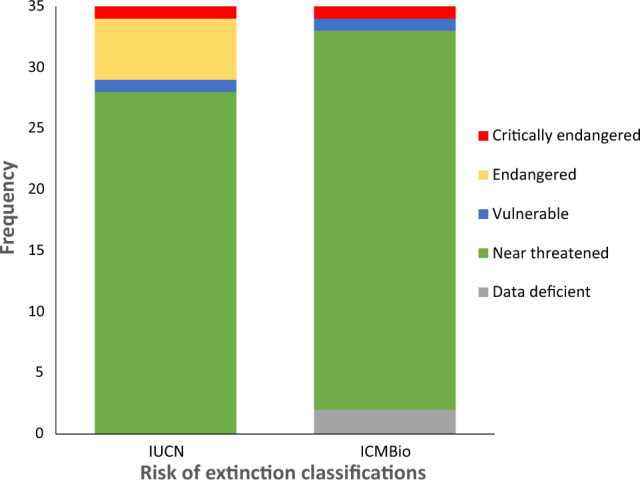
Bar chart showing the conservation classification of species identified in products at time of sampling (following the global IUCN Red List status in 2020 [35], and ICMBio, 2018 [7])

Of the “cação”products, 20% belonged to species threatened with extinction on the 2020 IUCN red list, including two vulnerable, three endangered and one critically endangered species (Table 1; Fig. 3).

**Table 1:** Species identified and their IUCN and ICMBio extinction risk classifications at the time of sampling (IUCN [35]; ICMBio [7]) and their current status

Species	Common name	IUCN (2020) [35]	IUCN (2023)	ICMBio (2020) [7]	ICMBio (2023) [42]
*Carcharhinus falciformis*	Silky shark	Vulnerable	Vulnerable	Near threatened	Critically endangered
*Carcharhinus signatus*	Night shark	Endangered	Endangered	Vulnerable	Endangered
*Carcharias taurus*	Sand tiger shark	Vulnerable (globally) Critically endangered (SW Atlantic population)	Critically endangered	Critically endangered	Critically endangered
*Isurus oxyrinchus*	Shortfin mako shark	Endangered	Endangered	Near threatened	Critically endangered
*Isurus paucus*	Longfin mako shark	Endangered	Endangered	Data deficient	Data deficient
*Prionace glauca*	Blue shark	Near threatened	Near threatened	Near threatened	Near threatened

**Fig. 4:**
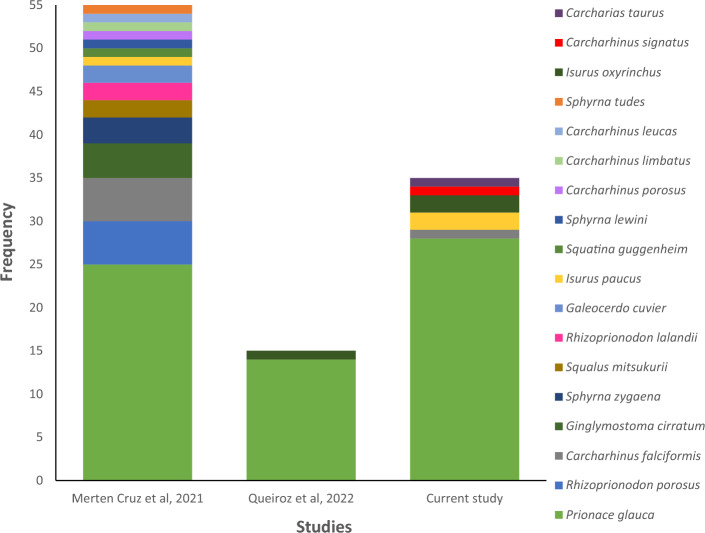
Bar chart of shark species commercialisation pre-pandemic and during Covid-19 lockdown

To explore the impact of COVID-19 lockdown on patterns of species commercialisation, a comparison was made between this study and two DNA barcode investigations conducted pre-pandemic [23, 36]. No significant difference between the species traded pre-pandemic and during lockdown was demonstrated. (ANOSIM p = 0.828 and p = 0.965, respectively) (Fig. 4).

## Discussion

The most striking result is that a fifth (20%) of the samples belonged to species threatened with extinction on the IUCN red list at the time of sampling, with *C. signatus*, *I. oxyrinchus* and *I. paucus* classified as endangered, while *C. taurus* was considered critically endangered locally. This finding is consistent with the results of previous studies that also identified these species being traded in Brazil [12, 20, 24–26].

Blue shark was by far the most prevalent species identified in products (80%) included in this study. Other Brazilian studies have also identified *P. glauca* as the most commonly sold species, accounting for between 24% [12] and 93% [36] of products. The result is consistent with the fact that blue shark represents 49–90% of the total pelagic sharks captured by commercial longline fisheries [9, 38], making this species the most common and widely traded shark in Brazil and internationally [24, 39]. Furthermore, *P. glauca* accounts for most of the shark carcasses being imported by Brazil [4], which ensures a year-round supply of shark meat [9, 40]. Although blue sharks are classified as near threatened by the IUCN [41] and ICMBio [7, 42], there has been growing concern for this species’ global conservation status due to overexploitation [38].

Shortfin and longfin mako were identified in two samples each, these species were reclassified as endangered by the IUCN in 2019 [43, 44]. In Brazil, *I. oxyrinchus* is currently listed as Critically Endangered, while I. paucus is classed as data deficient [42]. Mako sharks are highly prized for their fins and meat [40, 45] and, therefore, are of economic value to fisheries in Brazil. Moreover, after blue shark, shortfin mako is the most caught and reported shark in longline fisheries [46, 47]. Conversely, longfin mako sharks are only sporadically recorded as caught by Brazilian longline fisheries and are frequently grouped with *I. oxyrinchus* and other shark species in fisheries monitoring data [48]. Mako sharks have rarely been identified in previous barcode investigations of shark products in Brazil [23, 25, 36]. These results suggest that mako sharks may be a more significant component of Brazilian shark meat trade than previously thought, including in the region of São Paulo.

Perhaps the most surprising result was the identification of a sample as *C. taurus.* This finding is corroborated by previous work [24], which also reported sand tiger shark amongst the products analysed, despite records showing that only a few individuals of this species are landed every year in Brazil [49]. Sand tiger sharks are considered critically endangered [7, 42] in Brazil, and consequently, their commercialisation is banned. Therefore, this study showed that prohibited species are being traded under the label “cação” in São Paulo State. The global population of sand tiger sharks has only recently been upgraded from vulnerable to critically endangered [50] and as a result their commercialisation should be even more closely monitored.

The remaining samples were all identified as requiem sharks. One product was identified as *C. signatus*, a species which has been targeted in semi-pelagic fisheries since the 1990’s, [51] and is prohibited from being landed in Brazil. Previous studies have also reported night sharks amongst their samples [22, 25, 26]. The identification of another sample as *C. falciformis* in this study also supports the findings of previous research that vulnerable silky sharks were being traded in markets in Brazil [12, 24]. In fact, silky sharks have been particularly frequent in investigations conducted in the coastal regions of São Paulo State [26], suggesting that *C. falciformis* might be more commonly traded in this State.

When the samples were collected in May and June 2020, the State of São Paulo was experiencing its first wave of COVID-19 and the government instructed people to stay indoors, resulting in decreased fishing efforts and catch rates during this period [52]. It is possible that a reduction in monitoring and enforcement during the COVID-19 lockdown might also have increased the trade of endangered shark species, potentially explaining the sale of threatened and prohibited species identified here. However, comparison to the results of Merten Cruz et al. [23] and Queiroz et al. [36] gathered before the lockdown did not show a significant difference in the species traded. Perhaps reduced fishing effort during lockdown had little effect on bycatch, which accounts for much of the shark fishery, or there were minimal changes to fisheries enforcement during the lockdown. Despite the lack of a significant result, one striking difference is how much blue shark dominated the results here, at much higher proportions than most studies in Brazil, which could reflect an impact of the lockdown. It is suggested that blue shark likely originates from other countries, ensuring supplies of shark meat despite any local limitations on captures during lockdown. Brazil is a significant importer of shark meat, of which blue shark dominates the market, and the use of frozen products (perhaps even those captured before lockdown) could also have ensured continuous supply. While 60% of all products analysed in this study were collected fresh/unfrozen, it is difficult to ascertain whether these had been previously frozen and defrosted at retail chain. In fact, discussion with retailers during collection suggested that most sharks fished by longline fishing vessels in Brazilian waters are immediately frozen and sellers are known to defrost the meat and trade it as fresh to customers.

In the present research, restrictions of lockdown significantly complicated the collection of samples, limiting the number that could be collected. Other larger-scale investigations of shark products in Brazil, conducted prior to the COVID-19 pandemic, have also revealed similar sharks being traded, suggesting sales of species threatened with extinction is widespread regardless of lockdown [22, 25].

## Conclusion

This study demonstrated that endangered and prohibited species are being traded in Brazil under the label “cação”. This is a prime example of the type of ‘umbrella’ sales term frequently used in fisheries where many species, often of varied conservation concern, are labelled with the same designation [25, 53]. The use of the term “cação” can prevent accurate monitoring of domestic and imported fisheries products, while also hindering customers from making an informed decision [10, 19]. Improved species-level monitoring of seafood products [9, 22], alongside programmes to educate the public that “cação”is a term for shark meat [17], are key in preventing the ongoing exploitation of protected elasmobranchs in Brazil [10, 24]. Perhaps surprisingly, the comparison of species commercialisation before and during COVID-19 in São Paulo State did not demonstrate a significant difference, suggesting lockdown did not affect patterns of sale. Further investigations with an extended data collection period, higher number of samples and in-depth interviews with fishermen in this region, as well as other Brazilian States, are needed to provide more comprehensive evidence on shark meat trade in Brazil.

### Supplementary Information

Below is the link to the electronic supplementary material.

Supplementary file1 (XLSX 142 KB)

## Data Availability

The data presented in this study are available in supplementary material submitted with this manuscript.
